# A new species of jumping spider *Neonella* Gertsch, with notes on the genus and male identification key (Araneae, Salticidae)

**DOI:** 10.3897/zookeys.532.6078

**Published:** 2015-11-05

**Authors:** Gonzalo D. Rubio, Carina I. Argañaraz, Raquel M. Gleiser

**Affiliations:** 1 Instituto de Biología Subtropical, Universidad Nacional de Misiones (IBS, CONICET-UNaM), Puerto Iguazú, Misiones, Argentina; 2 Centro de Relevamiento y Evaluación de Recursos Agrícolas y Naturales (CREAN-IMBIV, CONICET-UNC), Ciudad de Córdoba, Argentina; 3 Cátedra de Ecología, Facultad de Ciencias Exactas, Físicas y Naturales, Universidad Nacional de Córdoba, Córdoba, Argentina

**Keywords:** Argentina, dichotomous key, neotropical, salticids

## Abstract

The American genus *Neonella* Gertsch, 1936 consists of very small jumping spiders whose biology is not well known. The genus currently includes eleven valid species, of which eight are known from both sexes and two are only known from one sex. This paper describes and illustrates a new species *Neonella acostae* **sp. n.**, demonstrates male palpal variation in *Neonella montana* Galiano, 1988, and provides some information on the ecology of three sympatric species. New records of *Neonella montana* and *Neonella minuta* Galiano, 1965 are reported. Because the previously described species of *Neonella* were well illustrated and diagnosed, a dichotomous key to males is given along with genital illustrations of both sexes for all known species.

## Introduction

The American genus *Neonella* Gertsch currently includes eleven valid species (Metzner 2015, WSC 2015, Ott et al. 2015), of which eight are known from both sexes and two are known from only one sex (*sensu* Prószyński 2015, updated in Ott et al. 2015). *Neonella vinnula* Gertsch, 1936, from the United States, was the first species described for the genus, and is the type species by monotypy. Subsequently, Galiano (1965, 1988, 1998) carried out the largest contributions to the genus and described eight species from Latin America: *Neonella minuta* Galiano, 1965; *Neonella antillana* Galiano, 1988; *Neonella lubrica* Galiano, 1988; *Neonella montana* Galiano, 1988; *Neonella nana* Galiano, 1988; *Neonella cabana* Galiano, 1998; *Neonella colalao* Galiano, 1998, and *Neonella mayaguez* Galiano, 1998. More recently, *Neonella camillae* Edwards, 2003 was described from Florida, USA. In addition, two Brazilian species were recently described, *Neonella salafraria* Ruiz & Brescovit, 2004 and *Neonella noronha* Ruiz, Brescovit & Freitas, 2007. The latest contribution to the genus was carried out by Ott et al. (2015), in which *Neonella cabana* was synonymized with *Neonella montana*.

*Neonella* jumping spiders are very small and easily unnoticed. The females are usually less than 2 mm in body length and the males are even smaller (Galiano 1998). This genus is similar to *Neon* Simon, 1876 (another genus of small jumping spiders) but can be distinguished by: 1) the absence of fovea; 2) the presence of abdominal scutum in males; and 3) the epigynal openings inside funnel-like atria (see Galiano 1988; and more detail in Gertsch 1936). The shorter distal embolus with thick tip is no longer considered a diagnostic character after Edwards (2003) described *Neonella camillae*, the first *Neonella* that has long and twisted embolus (“a retrolateral spiral with a proximal embolar disk”; see Edwards 2003, Fig. 5), and this kind of coiled retrolateral embolus was subsequently described for *Neonella salafraria* (see Ruiz and Brescovit 2004, Fig. 3) and *Neonella noronha* (see Ruiz et al. 2007, Fig. 13).

Recent phylogenetic analyses suggest that *Neonella* belongs to the subfamily Euophryinae, and falls within a clade with the Neotropical genera *Ecuadattus* Zhang & Maddison, 2012, *Belliena* Simon, 1902 and *Ilargus* Simon, 1901 (Zhang and Maddison 2013, 2015). Morphological characters indicate *Neonella* may be most closely related to *Darwinneon* Cutler, 1971 (not included in phylogenetic analyses), both of which are very small jumping spiders usually with a distinctive proximal tegular lobe (TL) and short RTA on male palp, and short and wide copulatory duct (Zhang and Maddison 2015).

The biology of these species is not well known. They have been found on the ground, e.g., *Neonella lubrica* and *Neonella nana* inhabiting leaf litter or underneath and in rotten wood (Galiano 1988), *Neonella camillae* in Australian pine litter no more than one cm in depth (Edwards 2003), *Neonella minuta* on grassland up to 40 cm high (Galiano 1965) and *Neonella montana* under small rocks (Galiano 1988). Probably their poor biological and taxonomic knowledge are due to their hidden habitat and the small size of individuals. As a result of an ecological study in Córdoba city, Argentina, several specimens of both sexes of three species of *Neonella* were collected: *Neonella minuta*, *Neonella montana* and an undescribed species. In this paper we describe and illustrate the new species, which we name *Neonella acostae* sp. n., show a variation of the male palp in *Neonella montana*, and provide some information on their ecology. Because the previously described species of *Neonella* were well illustrated and diagnosed, a dichotomous key for males to all known species is also given with this contribution.

## Material and methods

**Figure 1.**
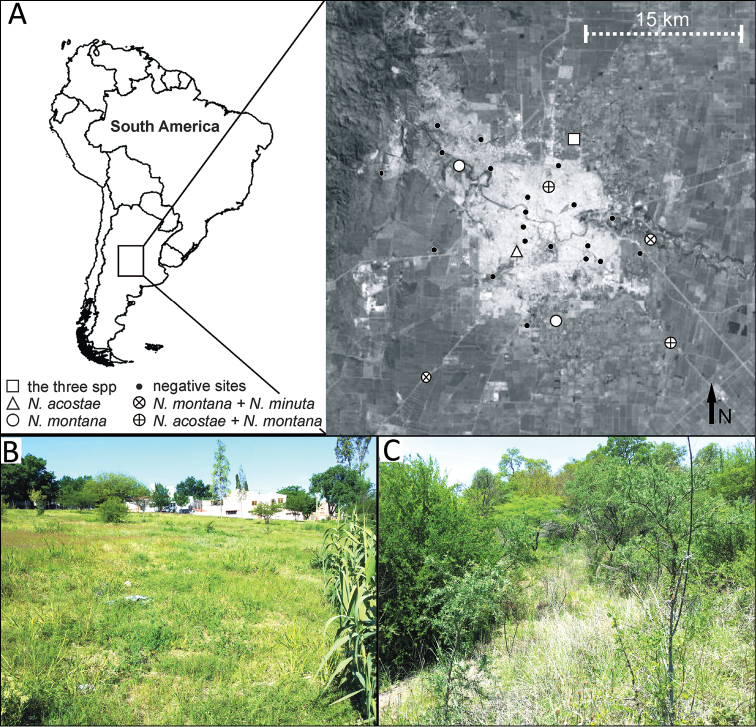
**A** Sampling location, positives sites for *Neonella* species and their distribution. Key: black circle = negative sites (further localities that were sampled but that did not yield *Neonella* spp.); white circle = *Neonella montana*; white triangle = *Neonella acostae*; white circle with a cross = *Neonella acostae* and *Neonella montana*; white circle with a X = *Neonella montana* and *Neonella minuta*; white squares = *Neonella acostae*, *Neonella minuta*, and *Neonella montana* **B** Typical location/habitat for *Neonella acostae* **C** Typical location/habitat for *Neonella montana*.

Specimens were collected in different sites in Córdoba city (central Argentina), using a Garden-Vacuum method to suck spiders from the vegetation (for details on the method, see Rubio and González 2010). We collected on thirty sites around Córdoba city, in urban and peri-urban habitats (in November 2013, springtime), from which nine sites provided *Neonella* species (Fig. 1A). Sites that were positive for *Neonella* were re-sampled the following February-March 2014 (summertime) and July-August 2014 (wintertime). The study area is located within the Espinal ecoregion (Brown et al. 2006), a thorny deciduous shrubland forest (Fund 2014), but has been historically subjected to intense anthropogenic disturbance and modifications (including deforestation, urbanization and agriculture). The sampling sites ranged from forest remnants to urban parks (Fig. 1B, C).

Description formats and morphological terms follow Ruiz and Brescovit (2004), Zhang and Maddison (2015), and Ramírez (2014). Female epigynum was dissected and cleared in clove oil to study the internal structures as in Levi (1965); the male bulb was similarly prepared. Temporary preparations were examined using a Leica DM500 compound microscope and a Leica M60 stereomicroscope. All measurements are in millimeters, were made with an ocular micrometer, and were measured as in Galiano (1963: 275). Leg measurements are shown as total length (femur, patella and tibia, metatarsus, tarsus). Specimens examined are deposited at the arachnological collections of: Museo Argentino de Ciencias Naturales “Bernardino Rivadavia”, Buenos Aires (MACN-Ar, C. Scioscia), Instituto de Biología Subtropical, Misiones (IBSI-Ara, G. Rubio) and Centro de Relevamiento y Evaluación de Recursos Agrícolas y Naturales, Córdoba (CREAN, C. Argañaraz).

Drawings in Figure 4 were modified from the following original sources: Edwards 2003 (Fig. 4A); Ruiz and Brescovit 2004 (Fig. 4B); Ruiz et al. 2007 (Fig. 4C); Galiano 1988, 1998 (Fig. 4E, I); Galiano 1998 (Fig. 4F, L); Galiano 1988, Ruiz et al. 2007, Zhang and Maddison 2015 (Fig. 4G); Galiano 1965, 1988 (Fig. 4H); Galiano 1988 (Fig. 4J, K).

The three species were collected together or at different locations. In order to explore the strength of the association or the degree to which two species occur jointly in a number of locations, Cole´s index (1949) was utilized. This association coefficient has been used in various applications over animals and plant ecology (Warrens 2008). The index was constructed by 2 × 2 contingency tables and χ^2^. A site was considered positive when a species was detected at least once either in the spring or the summer sampling (n = 9 sites). Significant associations could indicate interspecific interactions or similar responses to the same environment (Soosairaj et al. 2005).

Abbreviations used: ALE = anterior lateral eye; AME = anterior median eye; CD = copulatory duct; CO = copulatory opening; DS = dorsal scutum; E = embolus; EB = embolus base; FD = fertilization duct; MS = median septum; PA = patellar apophysis; PLE = posterior lateral eye; PME = posterior median eye (the smaller); LE = lamella of embolus; S = spermatheca; PSPL = prolateral spermophore loop; RSPL = retrolateral spermophore loop; RTA = retrolateral tibial apophysis; SP = spermophore; TL = tegular lobe; W = window of epigynum.

## Results

### Taxonomy Family Salticidae Blackwall, 1841 Subfamily Euophryinae Simon, 1901 Genus *Neonella* Gertsch, 1936

#### Neonella acostae sp. n.

Taxon classificationAnimaliaAraneaeSalticidae

http://zoobank.org/5925FF00-8AAD-4B19-B948-72334CA4EFAB

Figs 1A, B ; 2 ; 4D

##### Type material.

Holotype ♂ (MACN-Ar 34509) from near Toledo (31°32'10.54"S, 64°1'43.97"W; 381 m asl), Córdoba province, Argentina, 24.XI.2013, C.I. Argañaraz leg. Paratypes: 1 ♂ and 1 ♀ (IBSI-Ara 00242) and 1 ♀ (MACN-Ar 34510) from Ciudad de Córdoba (31°22'27.67"S, 64°10'42.70"W; 430 m asl), Córdoba province, Argentina, 23.XI.2013, C.I. Argañaraz leg.

##### Other material examined.

ARGENTINA: Córdoba: Ciudad de Córdoba, site 1 (31°22'27.67"S, 64°10'42.70"W; 430 m asl), 15.III.2014, C.I. Argañaraz & R.M. Gleiser leg., 1 ♂ (CREAN, tissue sample [tiss.s.] CIA 010); site 2 (31°26'6.13"S, 64°12'47.42"W; 441 m asl), 21.XI.2013, C.I. Argañaraz leg., 2 ♂ (CREAN); site 3 (31°20'18.24"S, 64°9'30.97"W; 438 m asl), 15.III.2014, C.I. Argañaraz & R.M. Gleiser leg., 1 ♀ (CREAN, tiss.s. CIA 008); near Toledo (31°32'10.54"S, 64°1'43.97"W; 381 m asl), 24.XI.2013, C.I. Argañaraz leg., 2 ♀ (CREAN).

##### Diagnosis.

**Figure 2.**
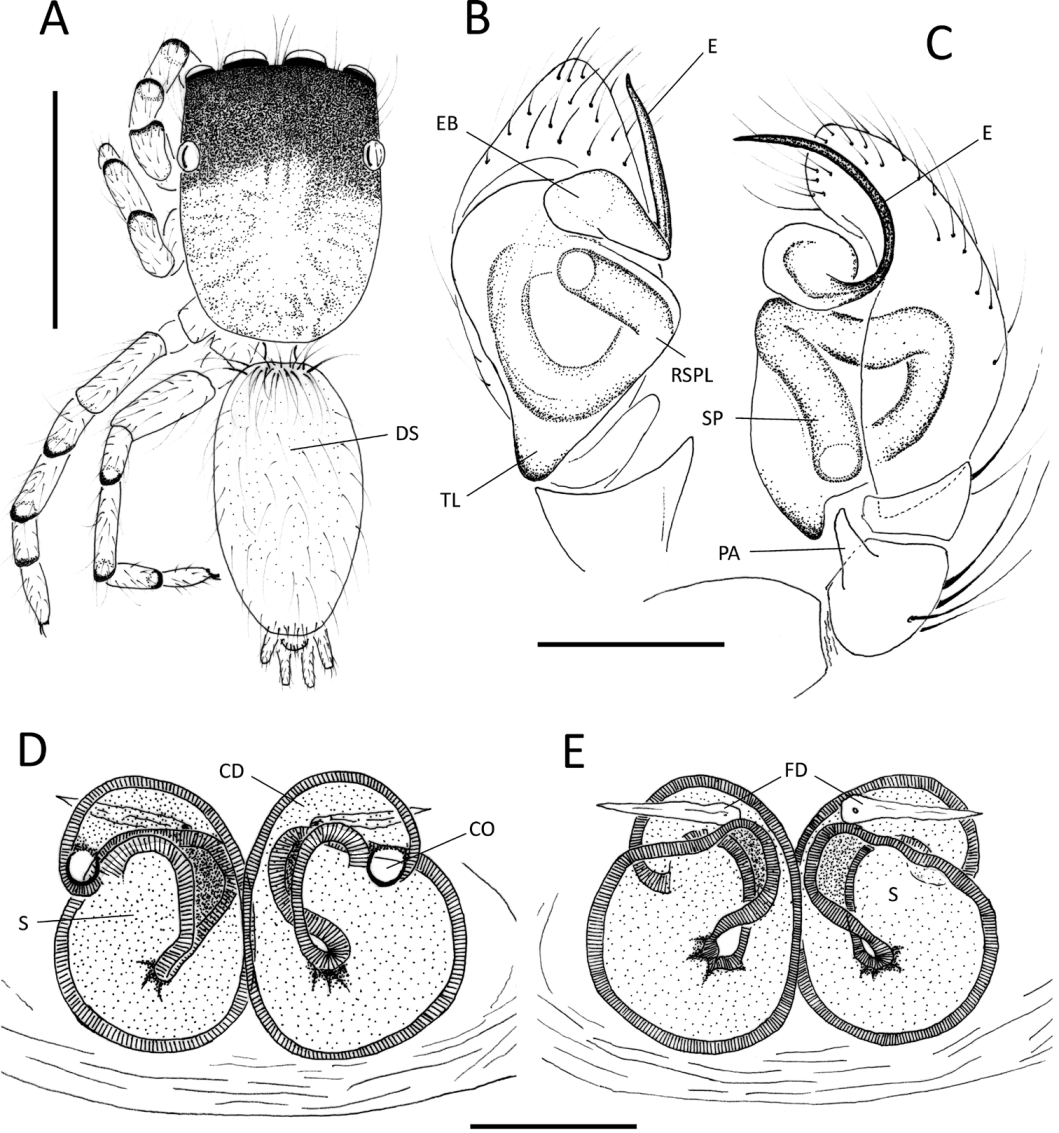
*Neonella acostae* sp. n.; **A–C** male (holotype) **A** dorsal habitus **B, C** male palp in ventral (**B**) and retrolateral (**C**) view **D, E** female (IBSI-Ara 00242) epigynum in ventral (**D**) and dorsal (**E**) view. (EB = embolus base; CD = copulatory duct; CO = copulatory opening; DS = dorsal scutum; E = embolus; FD = fertilization duct; PA = patellar apophysis; S = spermatheca; RSPL = retrolateral spermophore loop; SP = spermophore; TL = tegular lobe). Scale bars: 0.5 mm (**A**); 0.1 mm (**B, C**); 0.05 mm (**D, E**).

Males of *Neonella acostae* are similar to those of *Neonella camillae* and *Neonella noronha* in the coiled (semi-spiral) embolus (E), but can be distinguished from those and others with long spiral embolus by having only one patellar apophysis (PA) of palp (Fig. 2C; compare among Figs 4A–D). Furthermore, males differ from *Neonella noronha* in the shorter embolus. Females also resemble *Neonella noronha* and *Neonella salafraria* in having two small, round, simple copulatory openings (CO), but can be distinguished by having them more laterally placed and with different course of the copulatory ducts (CD) (Fig. 2D, E; compare with Figs 14, 15 in Ruiz et al. 2007 and Figs 4, 5 in Ruiz and Brescovit 2004).

##### Description.

Male holotype (Fig. 2A–C). Total length: 1.27. Carapace 0.62 long, 0.47 wide, 0.25 high; abdomen 0.62 long, 0.36 wide. Eye sizes: AME 0.12, ALE 0.087, PME 0.026, PLE 0.087. Ocular quadrangle 0.31 long. Anterior eye row 0.45 wide, posterior 0.47 wide. Clypeus height 0.017. Chelicerae with two very tiny promarginal teeth, hard to see; retromarginal teeth inconspicuous. Sternum longer (0.30) than wide (0.23). Leg measurements: I 0.78 (0.26, 0.25, 0.12, 0.14); II 0.70 (0.20, 0.25, 0.12, 0.12); III 0.86 (0.27, 0.29, 0.12, 0.17); IV 1.00 (0.31, 0.32, 0.17, 0.18). Carapace yellow with black spots, uniformly distributed; cephalic region darker to black, covered by white hairs. Thoracic region slightly lighter. Clypeus very low. Chelicerae tiny, yellow, grayish brown proximally. Legs pale yellow, with dark rings around the distal ends of the patella, tibia and metatarsus. Sternum and labium pale yellow. Palp (Fig. 2B, C): dark brown to black; cymbium brown, distally darker. Patella with a pointed retrolateroventral apophysis (PA). Copulatory bulb brown, with tegular lobe (TL) and conspicuous embolus base (EB). Embolus long (E), with a retrolateral half spiral (Fig. 2C). Abdomen pale yellow, uniformly covered with small black hairs; with an inconspicuous small thin dorsal abdominal scutum (DS). Spinnerets pale yellow. Variation (n=5): none apparent.

Female paratype (IBSI-Ara 00242) (Fig. 2D, E). Total length: 1.70. Carapace 0.75 long, 0.51 wide, 0.30 high; abdomen 0.87 long, 0.57 wide. Eye sizes: AME 0.14, ALE 0.075, PME 0.025, PLE 0.10. Ocular quadrangle 0.37 long. Anterior eye row 0.47 wide, posterior 0.50 wide. Clypeus height 0.012. Chelicerae as in male. Sternum longer (0.32) than wide (0.22). Leg measurements: I 0.93 (0.30, 0.32, 0.15, 0.15); II 0.86 (0.26, 0.30, 0.15, 0.15); III 0.99 (0.32, 0.32, 0.16, 0.17); IV 1.20 (0.37, 0.40, 0.22, 0.20). Carapace in general as in male, thoracic region slightly lighter. Clypeus very low. Chelicerae as in male, but light brown proximally. Legs, sternum and labium as in male. Palp yellow. Epigynum wider than long, with a thin translucent plate; two small copulatory openings (CO). Spermathecae tubular (S), connected to thick copulatory ducts (CD). Abdomen and spinnerets as in male; dorsal abdominal scutum absent. Variation (n=5): one female is more pigmented, with more dark spots on thoracic region.

##### Etymology.

The specific name is a Latinized patronym in honor of Dr. Luis E. Acosta, arachnologist of Universidad Nacional de Córdoba, who was major professor for the PhD of G.D.R. and advisor for the bachelor thesis of C.I.A.

##### Distribution.

Known only from Córdoba province (Fig. 1A): Ciudad de Córdoba and near Toledo, Argentina.

##### Sexual dimorphism.

Males and females differ only slightly in their somatic morphology. Females are slightly larger than males, mainly due to their larger abdomen. The carapace is somewhat more pigmented in males than in females.

#### Neonella montana

Taxon classificationAnimaliaAraneaeSalticidae

Galiano, 1988

Figs 1A, C ; 3 ; 4E

Neonella montana Galiano, 1988: 447, figs 14, 21 (holotype ♀ from ARGENTINA: Córdoba province, Cuesta Cura Brochero, deposited in MACN-Ar 8409, not examined); Ott et al. 2015: 586, figs 9‒12, 20‒25; Prószyński 2015; WSC 2015.
Neonella cabana Galiano, 1998: 15, figs 4‒6, 11, 12 (holotype ♂ from Cabana, Córdoba province, Argentina, not reexamined). Synonymized by Ott et al. (2015): 586.

##### Material examined (new records).

ARGENTINA: Córdoba: Ciudad de Córdoba, site 1 (31°22'27.67"S, 64°10'42.70"W; 430 m asl), 15.III.2014, C.I. Argañaraz & R.M. Gleiser leg., 1 ♀ (CREAN); site 3 (31°20'18.24"S, 64°9'30.97"W; 438 m asl), 23.XI.2013, C.I. Argañaraz leg., 1 ♂ and 1 ♀ (CREAN); Ciudad de Córdoba, site 4 (31°28'25.54"S, 64°11'17.44"W; 449 m asl), 21.XI.2013, C.I. Argañaraz leg., 1 ♂ and 1 ♀ (IBSI-Ara 00243); same loc., 20.III.2014, C.I. Argañaraz & R.M. Gleiser leg., 1 ♂ (MACN-Ar 34511); site 5 (31°21'41.23"S, 64°16'2.66"W; 451 m asl), 7.III.2014, C.I. Argañaraz & R.M. Gleiser leg., 1 ♂ (CREAN, tiss.s. CIA 001), 1 ♂ (CREAN, tiss.s. CIA 002), 1 ♂ (CREAN, tiss.s. CIA 003) and 1 ♀ (CREAN, tiss.s. CIA 004); near Comunidad Los Cedros (31°32'25.54"S, 64°18'14.69"W; 540 m asl), 28.XI.2013, C.I. Argañaraz leg., 1 ♂ (CREAN); near Ciudad de Córdoba (31°26'35.25"S, 64°3'48.09"W; 391 m asl), 29.XI.2013, C.I. Argañaraz leg., 3 ♂ and 1 ♀ (CREAN); same loc., 11/III/2014, C.I. Argañaraz & R.M. Gleiser leg., 3 ♂ and 3 ♀ (CREAN); Juarez Celman (31°15'13.69"S, 64°9'58.55"W; 500 m asl), 15.III.2014, C.I. Argañaraz & R.M. Gleiser leg., 4 ♂ and 1 ♀ (CREAN); near Toledo (31°32'10.54"S, 64°1'43.97"W; 381 m asl), 27.II.2014, C.I. Argañaraz & R.M. Gleiser leg., 1 ♀ (CREAN).

##### Note.

The holotype was requested for study but so far it is unavailable. However, we do not consider this a major drawback since in a recent contribution, Ott et al. (2015) synonymized *Neonella cabana* with *Neonella montana* based on specimens collected in southern Brazil, which had been noted as a possibility by Galiano (1998). In agreement with Ott and collaborators, we found that males of *Neonella montana* have variations in both somatic and reproductive structures. Therefore, an updated diagnosis including both sexes and an additional re-description of the male including the variation found in the palp of specimens from Argentina (near the type locality) are given below.

##### Diagnosis.

**Figure 3.**
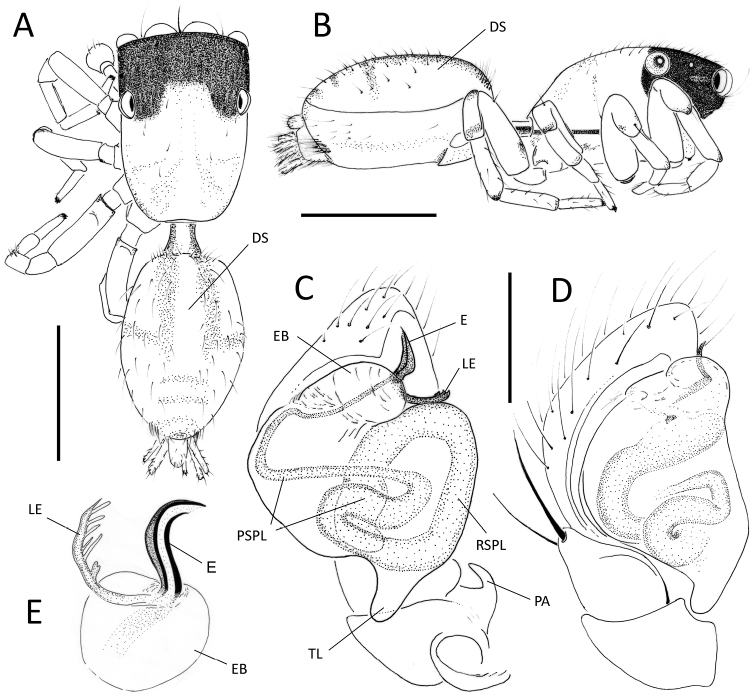
*Neonella montana* Galiano, 1988; **A–E** male (IBSI-Ara 00243); **A, B** habitus in dorsal (**A**) and lateral (**B**) view **C, D** male palp in ventral (**C**) and prolateral (**D**) view **E** detail of EB in dorsal view. (EB = embolus base; DS = dorsal scutum; E = embolus; PA = patellar apophysis; LE = lamella of embolus; PSPL = prolateral spermophore loop; RSPL = retrolateral spermophore loop; TL = tegular lobe). Scale bars: 0.5 mm (**A, B**); 0.09 mm (**C, D**).

**Figure 4.**
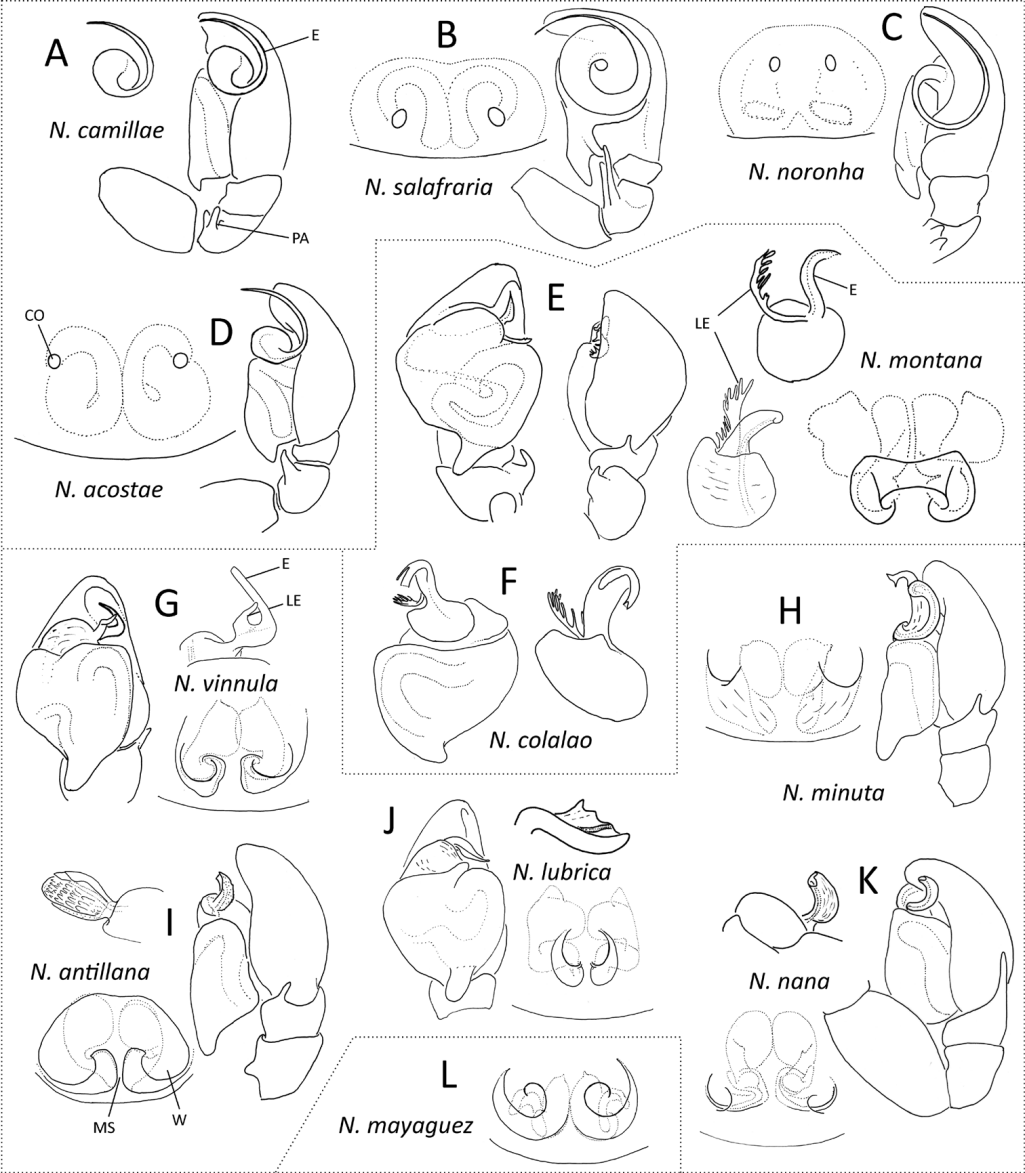
Schematic identification for species of *Neonella* Gertsch, 1936; **A–L** drawings modified from the original papers and descriptions (sources in method section). (CO = copulatory opening; E = embolus; MS = median septum; PA = patellar apophysis; LE = lamella of embolus; W = window of epigynum).

Males of *Neonella montana* are similar to those of *Neonella colalao* in sharing a comb-like, branched lamella of embolus (LE), but can be distinguished from this species by having non bifurcated terminal apex of the embolus (Fig. 3E; and Galiano (1998): Figs 11, 12, compare with Figs 7–10). Females of *Neonella montana* can be distinguished from the other species of *Neonella* by having only one opening on the epigynal plate, formed by a large, trapezoid atrium (Fig. 4E; and Galiano 1988: 447, Figs 14, 21).

##### Description.

Male from Ciudad de Córdoba (IBSI-Ara 00243) (Fig. 3). Total length: 1.47. Carapace 0.67 long, 0.47 wide, 0.32 high; abdomen 0.67 long, 0.44 wide. Eye sizes: AME 0.15, ALE 0.10, PME 0.025, PLE 0.090. Ocular quadrangle 0.35 long. Anterior eye row 0.50 wide, posterior 0.50 wide. Clypeus height 0.012. Teeth of chelicerae inconspicuous. Sternum longer (0.32) than wide (0.22). Leg measurements: I 0.96 (0.31, 0.32, 0.17, 0.15); II 0.80 (0.25, 0.27, 0.15, 0.12); III 0.96 (0.27, 0.32, 0.19, 0.17); IV 1.15 (0.32, 0.40, 0.22, 0.20). Carapace light brown with narrow black margins. Cephalic region black, covered by white hairs; thoracic region with a lighter longitudinal band. Clypeus very low. Chelicerae tiny, yellow, light brown proximally. Legs light brown, with blackish irregular bands on femurs side (pro and retrolateral), and blackish rings around the distal ends of the patella and tibia, and scarcely on metatarsus. Sternum and labium yellow. Palp (Fig. 3C–E): brown; cymbium yellow. Femur and patella black proximally, with a hook-shaped retrolateral apophysis (PA). Copulatory bulb light brown, with conspicuous tegular lobe (TL) and embolus base (EB). Embolus (E) short, with comb-like lamella (LE). Abdomen light brown, with a few scattered black hairs; with a pair of longitudinal dark stripes on the abdomen in anterior half, and the posterior half with chevrons. Dorsal abdomen completely covered with a scutum (DS). Spinnerets pale yellow. Variation (n=10): some specimens from Córdoba vary in thickness and shape of embolus and LE respectively; for comparison see Figure 4E; in addition, the blackish irregular bands of the femora may be less developed.

Female (Holotype, MACN-Ar 8409). The female is well illustrated and described in previous contributions: See Galiano (1988): 447 and illustration in Ott et al. (2015).

##### Distribution.

Central and southeast Argentina: in Córdoba (Fig. 1A) and Buenos Aires provinces, and southern Brazil: Rio Grande do Sul.

#### Neonella minuta

Taxon classificationAnimaliaAraneaeSalticidae

Galiano, 1965

Figs 1A ; 4H

Neonella minuta Galiano, 1965: 25, figs 1–8; Galiano 1988: 439, figs 17, 19; Ott et al. 2015: 585, figs 5‒8, 17‒19; Prószyński 2015; WSC 2015.

##### New records.

ARGENTINA: Córdoba: Juarez Celman (31°15'13.69"S, 64°9'58.55"W; 500 m asl), 23.XI.2013, C.I. Argañaraz leg., 1 ♀ (CREAN); same loc., 15.III.2014, C.I. Argañaraz & R.M. Gleiser leg., 1 ♀ (CREAN); near Ciudad de Córdoba (31°26'35.25"S, 64°3'48.09"W; 391 m asl), 29.XI.2013, C.I. Argañaraz leg., 2 ♀ (IBSI-Ara 00288); Ciudad de Córdoba, site 3 (31°20'18.24"S, 64°9'30.97"W; 438 m asl), 15.III.2014, C.I. Argañaraz & R.M. Gleiser leg., 1 ♀ (CREAN, tiss.s. CIA 009), 1 ♂ (CREAN), 1 ♂ and 1 ♀ (CREAN); near Comunidad Los Cedros (31°32'25.54"S, 64°18'14.69"W; 540 m asl), 26.II.2014, C.I. Argañaraz leg., 1 ♀ (CREAN).

##### Comments.

In a recent contribution, Ott et al. (2015) extend the distribution of *Neonella minuta* toward Rio Grande do Sul (Brazil), which was originally only known endemic to Buenos Aires (Argentina) by Galiano (1965). Our present work enhances the geographical distribution of this species, representing the westernmost record so far (Córdoba province, Central Argentina).

##### Ecology of the collected species.

The three species of *Neonella* were collected during the spring and the summer but were not detected in the winter samples. They were found in the lower strata of vegetation (0 to 35 cm), consisting mainly of grasses and forbs. *Neonella acostae* was collected both within the urban environment (Fig. 1A, B) and in more natural sites on the periphery of the city (Fig. 1A, C), while *Neonella montana* and *Neonella minuta* were mostly collected from more natural sites with dense vegetation on the city periphery (Fig. 1A, C). Based on Cole’s index (1949), *Neonella acostae* was negatively associated with *Neonella minuta* (-0.44 ± 0.42; mean association ± standard error) and *Neonella montana* (-0.13 ± 0.11), suggesting moderately dissimilar habitat disturbance tolerances because *Neonella acostae* was collected at a wider range of sites in terms of plant cover, or alternatively a moderate degree of interspecific competition because they occasionally occurred at the same site. Cumming and Wesolowska (2004) explained high Salticidae richness in small suburban areas as a result of strong host-plant associations. More detailed studies of microhabitat use should be carried out to confirm these explanations. *Neonella minuta* and *Neonella montana* were not significantly associated (0.1 ± 0.11), suggesting independent occurrences of the species.

### Provisional identification of species groups of *Neonella*

Known species of *Neonella* are more easily distinguished if based on the morphology of male organs; however, males with long and spiral embolus could have conspecificity with females having copulatory openings as two simple round holes and, apparently, without window of epigynum (W) or median septum (MS) (Fig. 4). All species included in the genus, 11 plus one described here, can be separated into two large main groups; 1) those with long spiral embolus: *Neonella camillae*, *Neonella salafraria*, *Neonella noronha*, and *Neonella acostae* (Fig. 4A–D), and 2) the remainder having short and generally more stout embolus. Within this latter group we can distinguish two subgroups; 2_a_) species having a visible branched lamella of embolus (LE): *Neonella montana* and *Neonella colalao* (Fig. 4E, F) and 2_b_) without such lamella or is very difficult to see, but in this case unbranched: *Neonella vinnula*, *Neonella minuta*, *Neonella antillana*, *Neonella lubrica*, and *Neonella nana* (Fig. 4G–K). Although it has been found that *Neonella noronha* has a tiny lamella of embolus (paraembolic projection *sensu* Ruiz et al. (2007)), this species has a long spiral embolus. *Neonella mayaguez* is known only from females (Fig. 4L).

The following key to species has some limitations because it is constructed based only on the males. Males have diagnostic characters which are much more apparent and applicable. On the other hand, in females, the diagnostic characters are mainly in the internal genitalia (ducts and spermathecae) in ventral and dorsal views (vulva), and these are more ambiguous. Therefore, we consider the need to complement this contribution with a comprehensive review of the genus to provide a key with both sexes in the future.

### Key to males of species of *Neonella*

**Table:** 

1	Copulatory bulb with long spiral embolus (Fig. 4A–D)	**2**
–	Copulatory bulb with short and generally stouter embolus (Fig. 4E–K)	**5**
2	Palpal patella with only one apophysis (Fig. 4D) (Argentina)	***Neonella acostae* auct**
–	Palpal patella with two or more apophyses (Fig. 4A–C)	**3**
3	Palpal patella with two long apophyses (Fig. 4B) (Brazil)	***Neonella salafraria***
–	Palpal patella with short apophyses (Fig. 4A, C)	**4**
4	Palpal patella with a spatulate apophysis, and two shorter apophyses (Fig. 4A) (USA)	***Neonella camillae***
–	Palpal patella with two short, triangular apophyses (Fig. 4C) (Brazil)	***Neonella noronha***
5	Copulatory bulb with a comb-like, branched lamella of embolus (Fig. 4E, F)	**6**
–	Copulatory bulb without such lamella or, if present, unbranched (Fig. 4G–K)	**7**
6	Embolic apex with two terminal rami (Fig. 4F) (Argentina)	***Neonella colalao***
–	Embolic apex non-bifurcated (Fig. 4E) (Argentina and Brazil)	***Neonella montana***
7	Copulatory bulb with a small lamella of embolus, as a separated structure (Fig. 4G) (USA)	***Neonella vinnula***
–	Copulatory bulb without such separated lamella of embolus (Fig. 4H–K)	**8**
8	Embolus retrolaterally directed, with an associated, laminar structure (Fig. 4J) (Paraguay)	***Neonella lubrica***
–	Copulatory bulb with the embolus apically directed (Fig. 4H, I, K)	**9**
9	Large, long embolus, with a dilated embolic apex (Fig. 4H) (Argentina)	***Neonella minuta***
–	Small, short, thick embolus, with a blunt embolic apex (Fig. 4I, K)	**10**
10	Short retrolateral tibial apophysis, surface of embolus conspicuously squamous (Fig. 4I) (Jamaica)	***Neonella antillana***
–	Retrolateral tibial apophysis longer, embolus without such surface (Fig. 4K) (Paraguay)	***Neonella nana***

## Supplementary Material

XML Treatment for Neonella acostae

XML Treatment for Neonella montana

XML Treatment for Neonella minuta
